# Planning Engaging, Remote, Synchronous Didactics in the COVID-19 Pandemic Era

**DOI:** 10.2196/25213

**Published:** 2021-05-11

**Authors:** Ronald Rivera, Jonathan Smart, Sangeeta Sakaria, Alisa Wray, Warren Wiechmann, Megan Boysen-Osborn, Shannon Toohey

**Affiliations:** 1 Department of Emergency Medicine University of California Irvine Medical Center Orange, CA United States

**Keywords:** distance education, videoconferencing, emergency medicine, teaching, learning, web-based lecture, medical education, technology, SARS-CoV-2, COVID-19

## Abstract

As part of the Accreditation Council for Graduate Medical Education requirements, residents must participate in structured didactic activities. Traditional didactics include lectures, grand rounds, simulations, case discussions, and other forms of in-person synchronous learning. The COVID-19 pandemic has made in-person activities less feasible, as many programs have been forced to transition to remote didactics. Educators must still achieve the goals and objectives of their didactic curriculum despite the new limitations on instructional strategies. There are several strategies that may be useful for organizing and creating a remote residency didactic curriculum. Educators must master new technology, be flexible and creative, and set rules of engagement for instructors and learners. Establishing best practices for remote didactics will result in successful, remote, synchronous didactics; reduce the impact of transitioning to a remote learning environment; and keep educators and learners safe as shelter-at-home orders remain in place.

## Introduction

Residency programs provide weekly or daily in-person, synchronous, didactic instruction to meet the Accreditation Council for Graduate Medical Education (ACGME) requirements for resident education [1]. Successful residency curricula are planned by using a thoughtful, systematic approach [2]. The ACGME recommends that educators establish appropriate goals and objectives for their curricula and decide on suitable instructional designs. Afterward, through program evaluation, educators use resident feedback, assessments, ACGME guidelines, graduate feedback, and specialty board certification requirements to make measured changes to their curricula [2]. As we transition to a more technologically advanced world, this approach has also been shown to work well in remote teaching [3].

In 2020, the implementation of shelter-in-place orders in response to the global COVID-19 pandemic, which was caused by the novel SARS-CoV-2, has tremendously disrupted regularly scheduled, in-person didactics for medical education programs [4-6]. Medical educators were compelled to transition from in-person lectures, simulations, and small groups to remote didactics [4,7,8]. Although many educators were familiar with accessing digital and prerecorded content for asynchronous learning, synchronous and remote didactics were less common prior to the COVID-19 pandemic [9]. Since 2008, the ACGME has allowed emergency medicine programs to use asynchronous-style learning to replace up to 20% of the required synchronous didactic time [1]. Approximately three-quarters of emergency medicine residencies have implemented web-based, self-directed learning with preselected resources [10]. Several small studies have suggested that web-based teaching modalities for residents and medical students may be equally as effective as in-person teaching modalities in various situations, including simulated patient encounters, ultrasound training, and procedural training [11-13]. However, remote didactics have traditionally only represented a small portion of didactics in graduate medical education. Educators previously chose from a potpourri of in-person instructional methods, such as lectures, labs, simulations, case discussions, team-based learning, and gamified didactics [1,14]. Now, educators must achieve the goals and objectives of their program by predominantly using remote instructional methods while maintaining the quality and integrity of their educational outcomes (generally defined by in-service scores and board scores) [1].

Popular videoconferencing platforms, such as Zoom, Microsoft Teams, StarLeaf, and WebEx, are the new classroom and meeting spaces. However, many remote “etiquette” items, such as keeping oneself muted or disabling cameras to conserve bandwidth, may hinder an instructor’s ability to interact with their audience and undermine the educational value of lectures. Previously effective methods of instruction, such as small-group instruction, team-based learning, gamification, and the use of audience response systems, may also be challenging to implement through these platforms; there may be technical disruptions due to a lack of familiarity with technology or due to connection issues [15,16]. It may also be difficult for learners to find a quiet, private place at home to attend didactics. Faculty members and students may experience additional distractions, such as childcare or other home responsibilities [17,18]. Furthermore, learners and faculty members may face additional stressors associated with COVID-19, such as mental health struggles, financial concerns, and housing disruptions. Such stressors may hinder their ability to attend didactics or focus [17,18]. In order to prevent interruptions in resident education or the decreased efficacy of resident education, it is important that we address these issues and find innovative methods for remotely conducting effective and engaging synchronous didactic sessions until in-person sessions can resume, the decision to hybridize curricula is made, or a transition to fully remote curricula becomes a reality [19].

After reviewing pre–COVID-19 pandemic literature on remote didactics and seeing a paucity of literature at the time of writing this paper, we herein suggest a list of best practices for planning and executing successful, remote, synchronous didactics during and beyond the COVID-19 pandemic. By building on the framework of Rubinger et al [20], which provides a theoretical approach to planning and executing remote conferences, our viewpoint paper aims to provide practical suggestions for planning multiple types of curricula and focuses on adapting existing, in-person lessons for immediate use while planning engaging lessons for future use [20].

## Update the Curriculum

Much of the success of asynchronous learning comes from an individual’s ability to work at their own pace and in accordance with their own schedule [21,22]. Synchronous didactics are often face-to-face meetings that require interactions, cooperation among groups, and responses to social cues that can present unique challenges during remote meetings. When transitioning to remote didactics, it is important to decide in advance which elements of an educator’s curriculum can be easily adapted to remote learning and implemented immediately and which elements require modification to be successfully integrated into a remote setting. There are specific ACGME guidelines that dictate the foundations of resident education [1]. These will likely be the initial focus of remote updates, since they are core requirements. Even educational activities that initially appear to be difficult to modify for remote learning, such as standardized patient cases, case-based role play, simulation, and skills training, may be implemented successfully [23,24].

Although the modality of didactics are changing during the transition from in-person didactics to remote didactics, curricular goals and objectives will still need to be met to ensure that learners continue to advance and didactic curricula comply with ACGME guidelines. The Kern 6-step curriculum model for planning traditional didactics remains applicable, but it has been successfully updated to meet the needs of remote learning [25,26]. The Council of Emergency Medicine Residency Directors Academic Assembly has already released guidelines for implementing and evaluating digital scholarship that may be used to plan ahead for these changes [27]. We recommend starting with the conversion of required didactics by using simple strategies like group lectures and audience participation. Afterward, additional time may be used to create more engaging and in-depth programs. Flexibility and creativity are critical for finding new ways to achieve a desired curriculum.

## Choose the Platform to Support the Activity

It is important to choose a platform or software that will support a program's specific needs, whether the intention is to host a didactic session that involves small-group breakout sessions, audience participation, or even simple large-group discussions. Institutional subscriptions may dictate the software that residency programs have access to or are allowed to use, but these institutional subscriptions also often provide additional features and functions that are not available in individual subscriptions. Although most platforms have similar functions, there may be unique features that make one platform more advantageous than others (Table S1 in Multimedia Appendix 1). For example, videoconferencing tools enable video-based dialogue between participants and instructors. Video livestreams allow instructors to broadcast their content; however, participants do not have access to interactive videos and are reliant on chat features or polls for interactions. Messaging platforms allow for real-time discussions among participants without the use of video. It is important to discuss institutional options with information technology groups to determine which platforms are accessible within an institution and which ones are compliant with an institution’s security policies. Consider using secondary applications and programs that can enhance one’s ability to present an engaging didactic session that promotes participation. Audience response tools are useful for creating interactive presentations that allow for audience participation (Table S2 in Multimedia Appendix 1). Additionally, familiarize the team with each program’s abilities and limitations and plan how to engage remote learners through the use of these tools. Be sure to also review a platform’s how-to videos and tutorials when planning a meeting in order to become familiar with and effectively incorporate interactive features without disruptions. The tables included in Multimedia Appendix 1 are not an exhaustive list of options. New programs are continuously being released, and platform developers are adding new features on an almost daily basis to support customers’ needs. This paper highlights some of the popular programs that we are familiar with and frequently use.

## Learning Environment

A key element of being an engaging presenter is the optimization of both the audio and visual components of a setup [16,28]. Keep in mind that much of this advice applies to all meeting participants, regardless of participants’ roles. In terms of audio quality, find a quiet space to host the presentation. Large rooms with bare walls and tiles will likely create distracting echoes, while small, carpeted rooms allow for clear sound quality [15]. Attempt to keep the amount of ambient noise to a minimum by alerting any housemates to the planned meeting or by leaving a sign on the door that tells housemates to not disturb the presenter. Avoid using high-demand internet streaming programs during the meeting to preserve bandwidth and prevent lag or a loss of connection. A clear, well-lit, and uncluttered video appearance is also important. Choose a space with minimal amounts of clutter or distractions in the background. Ideally, the camera should be placed just above eye level, which may require adjusting the chair or computer (eg, by using a stand or a stack of books) or using a free-moving camera [15,29]. When using multiple monitors, make sure to present from a front-facing monitor to allow for eye contact with the camera when presenting. Additionally, position the camera so that the speaker is seen from the chest up. This allows for a more natural view when showing any hand gestures. Everything that is needed for the meeting should be close to the presenter so that they can avoid standing up and moving around during the presentation [29]. Positioning the light sources in front of the speaker instead of behind the speaker will prevent backlight from obscuring the presenter’s image [15]. Avoid the use of multiple different light sources, as this may “wash out” the image if the light sources are not correctly positioned. Additionally, the use of direct light often results in a harsh or stark appearance. This may be counteracted by using a light filter attachment or by bouncing indirect light off of a wall [29].

## Technology

Once the meeting platform is chosen, ensure that the latest version of the software is downloaded and that there are no pending updates that will disturb the meeting. Use a computer rather than a smartphone or tablet to allow for large screen ratios [15]. Close any unnecessary background programs so that more computing memory is available. Turn on the “do not disturb” modes of the computer and surrounding devices that may interrupt the presentation [29]. Ensure that the program only shares the portion of the screen that participants should see and hide or close messaging services, emails, or other private information. Many experts suggest using headphones to avoid feedback loops from a computer’s microphone, which can detect meeting sounds. However, many new devices have technology that automatically filters out sounds from meetings [29]. When using headphones, consider using the computer’s microphone to achieve better sound quality. In our experience, computer microphones often provide better sound quality than headphone microphones, and professional microphones provide the best quality. Make sure all of the devices are powered and charged throughout the meeting [15]. When giving a presentation and using speaker notes, make sure to share the screen properly while still having access to the speaker notes. Additionally, be sure to have access to any other necessary tools while presenting, such as chat features, whiteboard features for annotations, and additional audience response programs that might be used during the presentation. Consider conducting a trial run with a friend or colleague to see how the setup appears on learners’ screens, so that adjustments can be made as needed [29]. For certain activities, it may be helpful to have a cohost during the meeting to help with moderating chat rooms, asking questions, providing answers, or conducting breakout rooms. It is also important to ensure that technology is appropriately set up in advance to avoid interruptions that may reduce teaching efficacy and learner engagement [28].

## Security

It is important to review the security options that are available on one’s videoconferencing platform. In the COVID-19 pandemic era and remote meetings, “Zoombombings” (unwelcome and vocal meeting guests) are a potential security threat [30]. Especially when discussing patient care for the purpose of quality improvement, it is essential that one’s videoconferencing platform has adequate security features, including encryption and meeting access control [31]. When creating a meeting, one should use a unique meeting ID instead of a repeated standard ID. This will limit a hacker’s ability to find the meeting. Meetings can be protected by a password or be based on invitation lists, which only allow certain participants to enter a meeting. Zoom offers a “Waiting Room” feature that allows hosts to approve participants before they can enter a meeting. When setting up a meeting, restrict screen sharing so that permission must be granted for participants to share their screens. Settings can also be changed to mute all participants upon entry, which often eliminates disruptions from late attendees. With regard to meetings that are disrupted by a participant or hacker, Zoom offers a “Put Everyone on Hold” feature that stops the video and audio feeds until the host turns them back on. The host can also remove disruptive participants from a meeting. We recommend activating the feature that will not allow removed participants to rejoin the meeting. Knowing how to appropriately secure the meeting is incredibly important to protecting the learning environment and improving the efficacy of didactics [28].

## Engagement

Based on an institution’s goals, set up specific rules for didactic engagement that can be distributed to participants in advance. Our didactic programs have a variety of faculty members, different postgraduate year (PGY) levels, and senior medical students. We recommend asking participants to change their on-screen name so that it is displayed as their first name, last name, and position (eg, “medical student 3,” “medical student 4,” “PGY1,” “PGY2,” “Fellow,” “Attending,” etc). This allows for the easy provision of assignments to small groups and allows lecturers to identify participants by their learning levels. Participants should be asked to keep their cameras on when they are in front of the computer (as a way to monitor participants’ engagement) and to only turn their cameras off when they need to momentarily step away. Keep in mind that some learners may need to turn their cameras off to improve streaming quality or for personal reasons (eg, a nursing mother). It is best to privately message participants when requesting them to turn on their cameras so that these exceptions can be discussed rather than publicly calling them out. Some institutions also encourage participants to list their gender pronouns (he/him, she/her, and they/their) to facilitate easy interactions with audience members who may not have their video stream activated. Microphones should be muted in large groups and unmuted during free-form discussions or in small groups. On some platforms, the meeting host can mute an individual or all participants with the click of a button. This is helpful in case someone forgets to mute or unmute themselves or if one’s sound becomes disruptive [29]. If a group chat function is available, remind participants that the main group chatroom should not be used for side discussions during a presentation; the group chatroom should be used to ask pertinent questions, make comments, or provide resources. Some platforms offer participants the ability to signal the speaker when they have questions with a “Raise Hand” button. Remind participants that when asking questions, there is often a keyboard shortcut key (eg, space bar, “M” button, etc) that temporarily unmutes the microphone while it is held down. This is perfect for asking questions in large group settings because the participant becomes muted again when they are done asking their question. Cohosts may help manage chatrooms or alert instructors to questions. Most platforms use a participant list to record attendance. Remind participants about whether lectures are to be recorded and inform them that all messages (including private messages) are logged.

## Large Groups

We separate large group activities (all participants are in a single remote space) from small group activities (participants are split into multiple interactive breakout rooms) when planning didactics. We found that it was easy to convert in-person sessions with large groups to remote sessions and that large group sessions were an ideal format for inviting distant or well-known speakers for whom an in-person lecture may not have previously been feasible. However, remote didactics in a large group setting can make audience engagement and participation difficult. Participants may be easily lost in the crowd, and instructors may feel as if they are speaking to an empty room. We recommend several methods for making these large group sessions more interactive. The simplest tool is the chat box, which allows instructors to ask questions and provide answers to participants. This feature works best when the instructor is looking for a single correct response, as numerous responses may quickly become unmanageable in this space. Some platforms offer a polling option that keeps participants’ answers organized in a way that is easy for both instructors and participants to visualize. Some software platforms also possess a whiteboard option that allows for on-screen annotation by audience members. This feature is especially useful for visual topics such as electrocardiograms and radiology images, as it provides learners with the ability to mark findings that they believe are important in real time for everyone to see. Even platforms that are traditionally used for messaging or posting, like Instagram, Twitter, and Facebook, can be used to disseminate interesting cases, radiographs, or electrocardiograms and conduct real-time assessments [32]. Audience response programs also provide unique audience engagement features that scale well for large groups (Table S2 in Multimedia Appendix 1). Such programs may be paired with resources such as Emergency Medicine Coach, Emergency Medicine Foundations, ECG Stampede, and other question banks to facilitate large-group participation.

## Small Groups

Successfully promoting the engagement of small groups requires more advanced planning than the planning required for other didactics. Based on the activity, divide participants into specific groups. This may take several minutes depending on the chosen platform. Didactics such as team-based learning or small group discussions often work best with an equal mix of students of various PGYs and medical students [33]. In many conferencing programs, the host can preassign breakout groups by using the email address that was used to create a participant’s account. To make this process more rapid, we found it helpful to create a web-based form in which residents entered their account email addresses (in case the account was created using a noninstitutional email). Creating group matrices for each specific group type in advance may help with making the uploading process easier. However, preassigning groups may not work or may prove to be time consuming in small residency programs or programs without protected time for face-to-face didactics in which residents attend conferences based on their work schedule. In this case, having the name and PGY in each participant’s screen handle allows the host to easily sort the participants as needed for each specific activity. This may be performed in the background during a large group lecture to limit the amount of lost time between activities. Ideally, groups should have 5-8 members and 1-2 leaders, if feasible [33]. Ultimately, it will be up to the group leader(s) to ensure that all participants are engaged, but this is no different from the expectations in face-to-face didactic sessions.

Standardized patient cases can be adapted and administered to small groups via videoconferencing platforms. Standardized patients can answer questions that are presented by the interviewer, physical exam maneuvers can be narrated by the interviewer, and findings can be presented by the instructor in real time. For example, after a verbal interview regarding the elements of a patient history, a learner can transition to the physical exam portion by saying, “I am now going to listen to the heart, what do I hear?” Afterward, the instructor can provide the pertinent positive and negative findings. This also works for case-based role play in small groups with instructor supervision and instruction. Simulation sessions can be remotely conducted in small groups after a small amount of advanced preparation. A simulation technician can prepare slides with pictures or videos of a patient monitor, electrocardiograms, imaging studies, and pertinent physical findings that will be shared by the facilitator. This is what would normally be done during in-person simulation sessions. The instructor is still able to act as the confederate or nurse while the technician shares their screen with the group. With even more preparation, skills training can also be remotely accomplished by sending kits with prearranged materials to learners by mail or having learners pick the items up from a central office. The learners will then have the training materials and be able to remotely follow a videoconference lesson in which an educator shares videos of how to use the materials and practice the skills intended. It is important to recognize that there may be a more time-intensive remote conversion for these types of synchronous didactics, and they can be difficult to administer without advance testing and practice.

## Interaction

As previously mentioned, large group sessions can be made more interactive by asking questions to the audience and allowing them to respond verbally or write responses with the chat feature. Blank slides can be inserted into presentations to act as a whiteboard for group annotations. Polls can be added regularly throughout the lecture to keep the audience involved or to ask relevant questions. Kahoot! offers presenters the ability to ask questions in a competitive quiz format, and the premium version allows for presentations with integrated questions. Ultrasound and procedure lectures can be enhanced by using multiple cameras that allow the audience to see an ultrasound screen or procedure and the presenter at the same time. Game show–style didactics, such as Jeopardy and Family Feud, can also be used in both large and small group settings to promote engagement. Consider combining gamified learning with escape room–type challenges or pick-a-pathway–style learning sessions for smaller groups. We have successfully done this with toxicology-related and nervous system disorder–related materials [34,35]. Participants in gamified education sessions rated their engagement with these types of activities much higher than those in other types of small group sessions [36-38]. Even using collaborative webspaces, like those provided in Google Forms and Microsoft Forms, can allow participants to perform team brainstorming, provide responses to questions, or analyze patient cases. These webspaces can add important elements of group participation to remote didactics and breakout sessions. We have even used collaborative webspaces to allow learners to ask questions and confidentially provide comments during sensitive or controversial lectures as a way to promote the freedom of discussion.

## Archival Methods

Many remote meeting platforms offer the ability to record lessons. Some platforms also have the ability to record the speaker and the shared screen at the same time and place them side by side in the video. These recordings are especially useful for creating free, open-access medical education materials if the institution chooses to publish them [39,40]. Sites such as YouTube, Instagram, and Facebook are excellent platforms for sharing lectures. Additionally, when creating an archive of lectures, any learners who cannot attend a session can refer back to the archive, thereby turning the synchronous learning activity into an asynchronous activity. Some technical experts also suggest using a smartphone to record a redundant copy of the audio during a didactic session so that it may be used to supplement any audio interruptions resulting from bandwidth issues [15]. iPhones have an app called Voice Memos and Android has an app called Voice Recorder; these apps can be used for audio recording purposes. Archived lectures can also be used as tools for recruiting prospective residents and medical students.

## Evaluation

Feedback is essential for evaluating educational programs and improving learner engagement [2]. During remote didactics, this should be no different. Services like Google Forms, Microsoft Forms, Survey Monkey, and Qualtrics can be used to create standardized evaluation forms that use Likert scales and prompt participants to share learning points from each activity in the same way that continuing medical education activities are evaluated [41-44]. This feedback is essential for promoting individual presenters’ engagement in the continuous quality improvement of their content and identifying areas for future faculty development [1,2]. At the program level, this feedback provides data about the effectiveness of didactic sessions and various modalities for remote didactics that are necessary for future curriculum planning.

## Asynchronous Learning

ACGME requirements allow residents to supplement their synchronous learning with asynchronous activities [1], and we recommend conducting prelearning and follow-up activities to promote knowledge retention. Prereading activities, which are associated with the “flipped classroom” curriculum style, can be used to prepare for small-group and team-based learning exercises [45]. The continuation of topic discussions through resident interest groups or mini fellowships can also be remotely achieved by video or email. Supplemental articles can be assigned, allowing learners to create summaries or discussion points with their mentors or education leaders. Follow-up cases, such as oral boards or simulations, can also be used to reinforce learning. Other options for asynchronous resources are high-quality educational blogs with content that mirrors residency curriculum topics, such as the Academic Life in Emergency Medicine’s Approved Instructional Resources Series [46]. Some board review sites and similar question bank sites allow for the selection of themed questions that can be assigned to learners as a supplemental activity. Do not forget to offer recorded lectures to learners who want to make up for a missed lecture or conference. Curating a variety of asynchronous learning options also helps learners identify resources and develop a sustainable strategy for their own self-directed and lifelong learning [47].

## Conclusions

The world is experiencing difficult times during the COVID-19 pandemic, which has changed how we personally and professionally interact with each other. Educators are at a unique crossroad; they must update their teaching strategies and accommodate remote learning sessions that are equally as effective as in-person sessions. By embracing technology and taking a creative approach to develop engaging, remote, didactic sessions, we can limit the interruption of resident learning. The lessons we learned from our experiences may even change the way we approach in-person learning in graduate medical education in the future.

## Appendix

Multimedia Appendix 1 Platforms, applications, and software adjuncts for remote synchronous education.

## Abbreviations

ACGME: Accreditation Council for Graduate Medical Education
PGY: postgraduate year
